# Synthesis, crystal structure and thermal properties of *catena*-poly[[bis­(4-methyl­pyridine)­nickel(II)]-di-μ-thio­cyanato], which shows an alternating all-*trans* and *cis–cis*–*trans*-coordination of the NiS_2_N^p^_2_N^t^_2_ octa­hedra (p = 4-methyl­pyridine, t = thio­cyanate)

**DOI:** 10.1107/S2056989024005887

**Published:** 2024-06-21

**Authors:** Christian Näther, Sebastian Mangelsen

**Affiliations:** a Institut für Anorganische Chemie, Universität Kiel, Max-Eyth.-Str. 2, 24118 Kiel, Germany; University of Aberdeen, United Kingdom

**Keywords:** synthesis, crystal structure, coordination polymer, chain structure, thermal properties, nickel thio­cyanate, 4-methyl­pyridine

## Abstract

The title compound, [Ni(NCS)_2_(C_6_H_7_N)_2_]_*n*_, was prepared by the reaction of Ni(NCS)_2_ with 4-methyl­pyridine in water. Its asymmetric unit consists of two crystallographically independent Ni^II^ cations, of which one is located on a twofold rotational axis whereas the second occupies a center of inversion, two independent thio­cyanate anions and two independent 4-methyl­pyridine co­ligands in general positions. Each Ni^II^ cation is octa­hedrally coordinated by two 4-methyl­pyridine coligands as well as two N- and two S-bonded thio­cyanate anions. One of the cations shows an all-*trans*, the other a *cis*–*cis*–*trans* configuration. The metal centers are linked by pairs of μ-1,3-bridging thio­cyanate anions into [101] chains. X-ray powder diffraction shows that a pure crystalline phase has been obtained and thermogravimetry coupled to differential thermoanalysis reveals that the title compound loses half of the 4-methyl­pyridine coligands and transforms into Ni(NCS)_2_(C_6_H_7_N). Nearly pure samples of this compound can be obtained by thermal annealing and a Rietveld refinement demonstrated that it is isotypic to its recently reported Cd analog [Neumann *et al.*, (2020 ▸). *CrystEngComm.***22**, 184–194] In its crystal structure, the metal cations are linked by one μ-1,3(*N*,*S*)- and one μ-1,3,3(*N*,*S*,*S*)-bridging thio­cyanate anion into single chains that condense *via* the μ-1,3,3(*N*,*S*,*S*)-bridging anionic ligands into double chains.

## Abstract 2

In the crystal structure of the title compound, Ni(NCS)_2_(C_6_H_7_N)_2_ (C_6_H_7_N = 4-methyl­pyridine), the Ni^II^ cations are in an octa­hedral coordination and are linked by pairs of anionic ligands into corrugated chains in which the cations show alternating all-*trans* and *cis*–*cis*–*trans* coordination geometries. Upon heating, the title compound transforms into Ni(NCS)_2_(C_6_H_7_N), which is isotypic to its Cd analog as proven by a Rietveld refinement.

## 1. Chemical context

**Figure 1:**
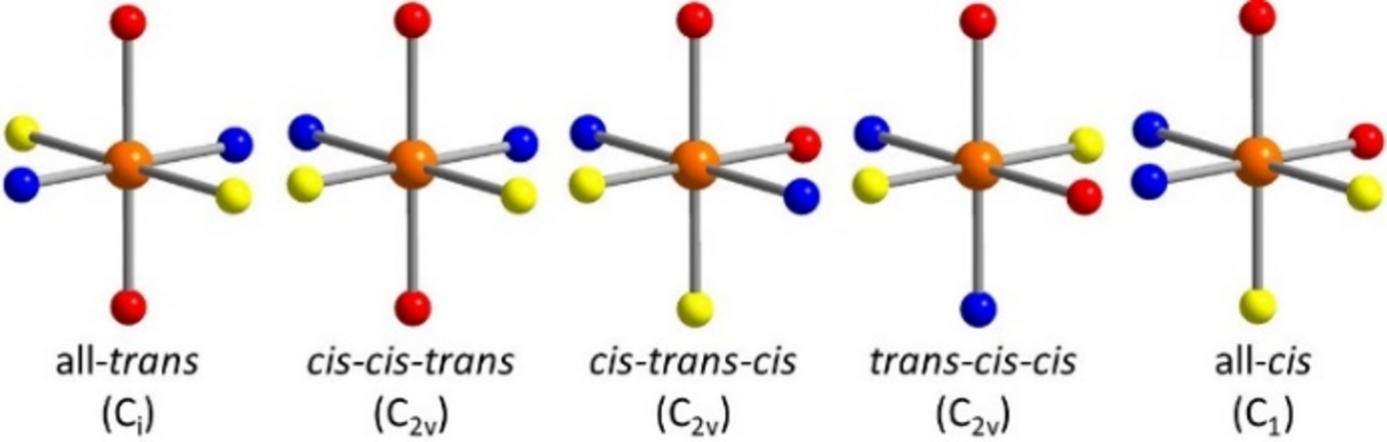
View of the different geometric isomers for an octa­hedral *MA*_2_*B*_2_*C*_2_ coordination with the corresponding notation and idealized point group. The N atoms of the neutral co-ligands are shown in red.

Coordination compounds based on transition-metal thio­cyanates are an inter­esting class of compounds because they show an extremely large structural variability that can also lead to different polymorphs and isomers (Wöhlert *et al.*, 2013 ▸; Neumann *et al.*, 2018 ▸; Jochim *et al.*, 2020 ▸). This can be traced back to the fact that this anionic ligand shows many coord­ination modes (terminal, μ-1,1- and μ-1,3-bridging) and that compared to, *e.g.* azides, the coordinating donor atoms are different. Moreover, in many cases an octa­hedral coordination of the metal center is observed and in such compounds the metal cations are usually linked by pairs of anionic ligands into chains. Chain compounds are usually formed with mono-coordinating neutral coligands, whereas bridging coligands lead to the formation of layers. Five different isomeric configurations exist for such an octa­hedral coordination, including all-*trans* and all-*cis* and three different *cis*–*cis*–*trans* configurations (Fig. 1 ▸). In the majority of compounds, an all-*trans* configuration is observed but there are also examples of compounds in which the all-*cis*, *cis*–*cis*–*trans*, *cis*–*trans*–*cis* or *trans*–*cis*–*cis* configurations are present (see *Database survey*).

The structural variability is further increased if such configurations alternate and in this context we have reported on compounds with the composition Ni(NCS)_2_(4-chloro­pyridine)_2_ [Cambridge Structural Database (CSD) refcodes UHUVIF and UHUVIF01; Jochim *et al.*, 2018 ▸] and Co(NCS)_2_(4-chloro­pyridine)_2_ (GIQQIJ and GIQQIJ01; Böhme *et al.*, 2020 ▸). For this compound, two different isomers were obtained, in both of which the Co^II^ cations are octa­hedrally coordinated and linked into chains by pairs of thio­cyanate anions. However, in one of these isomers an all-*trans* configuration is present, leading to the formation of linear chains, whereas in the second an alternating all-*trans* and *cis*–*cis*–*trans* configuration is observed, which leads to the formation of corrugated chains. Solvent-mediated conversion experiments show that the isomer with corrugated chains is more stable than that with linear chains, which is suprising because, as mentioned above, most compounds form linear chains (Jochim *et al.*, 2018 ▸; Böhme *et al.*, 2020 ▸). In this context, it is noted that not only the metal configuration can alternate, because we have prepared the first Co(NCS)_2_ coordination polymer with a linear chain structure in which an alternating fivefold and sixfold coordination is present (WEKVUH; Böhme *et al.*, 2022 ▸).
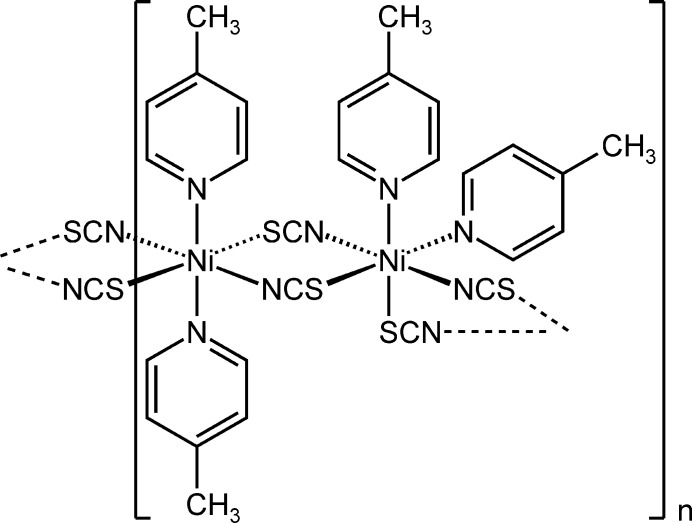


However, based on the results for the 4-chloro­pyridine compounds, we remembered the chloro–methyl exchange rule, which states that compounds with such ligands are very often structurally similar or even isotypic due to the fact that the van der Waals radius of a methyl group is similar to that of a chlorine atom (Desiraju & Sarma, 1986 ▸). Consequently, we assumed that similar isomers might be prepared with 4-methyl­pyridine, C_6_H_7_N. Therefore, Ni(NCS)_2_ was reacted with 4-methyl­pyridine, which leads to the formation of the title compound with the composition Ni(NCS)_2_(C_6_H_7_N)_2_, which is isotypic to the most stable isomer of Ni(NCS)_2_(4-chloro­pyridine)_2_ and Co(NCS)_2_(4-chloro­pyridine)_2_, which are already reported in the literature (Jochim *et al.*, 2018 ▸). We have not found any evidence for the formation of a metastable isomer with linear chains and 4-methyl­pyridine as ligand.

In this context it is mentioned that a compound with the stated composition Ni(NCS)_2_(C_6_H_7_N)_2_ is already reported in the CSD (QQQGJV; Solaculu *et al.*, 1974 ▸). This compound crystallizes in the ortho­rhom­bic space group *I*222 with the unusual value of *Z* = 18 formula units in the unit cell. Unfortunately, no atomic positions were presented and this entry is limited to unit-cell parameters, crystal system and space group. In fact, for this space group *Z* = 16 would be expected if two crystallographically independent formula units were present in the asymmetric unit, and in this case the volume for each non-hydrogen atom is calculated to be 23.2 Å^3^, which is a relatively high value and might point to some solvent mol­ecules being present. Therefore, from our point of view the existence of this crystalline form is at least questionable.

There is another entry in the CSD with this composition and for this structure atomic coordinates are available (ITMPNI; Lipowski & Andreetti, 1978 ▸). In this structure, each Ni^II^ cation is octa­hedrally coordinated by 4-methyl­pyridine coligands, two terminal N-bonded thio­cyanate anions as well as one S- and one N-bonding bridging thio­cyanate anion. Each two Ni^II^ cations are linked by pairs of μ-1,3-bridging anionic ligands into dinuclear complexes. Because the chemical composition is identical to that of the title compound, it might be denoted as an isomer.

## 2. Structural commentary

**Figure 2:**
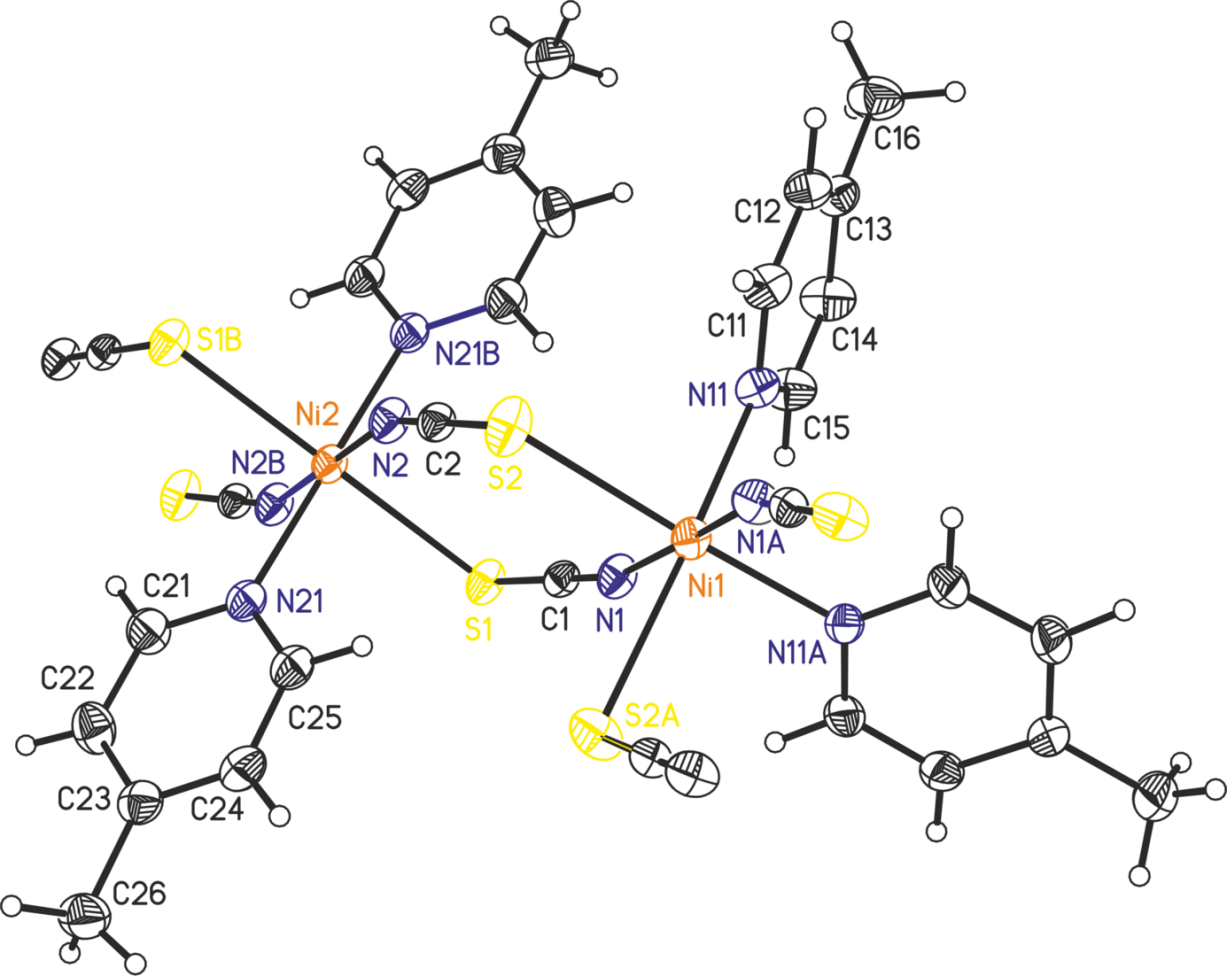
Crystal structure of the title compound with labeling and displacement ellipsoids drawn at the 50% probability level. Symmetry codes (i) −*x* + 1, *y*, −*z* + 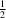; (ii) −*x* + 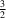, −*y* + 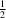, −*z* + 1.

**Figure 3:**
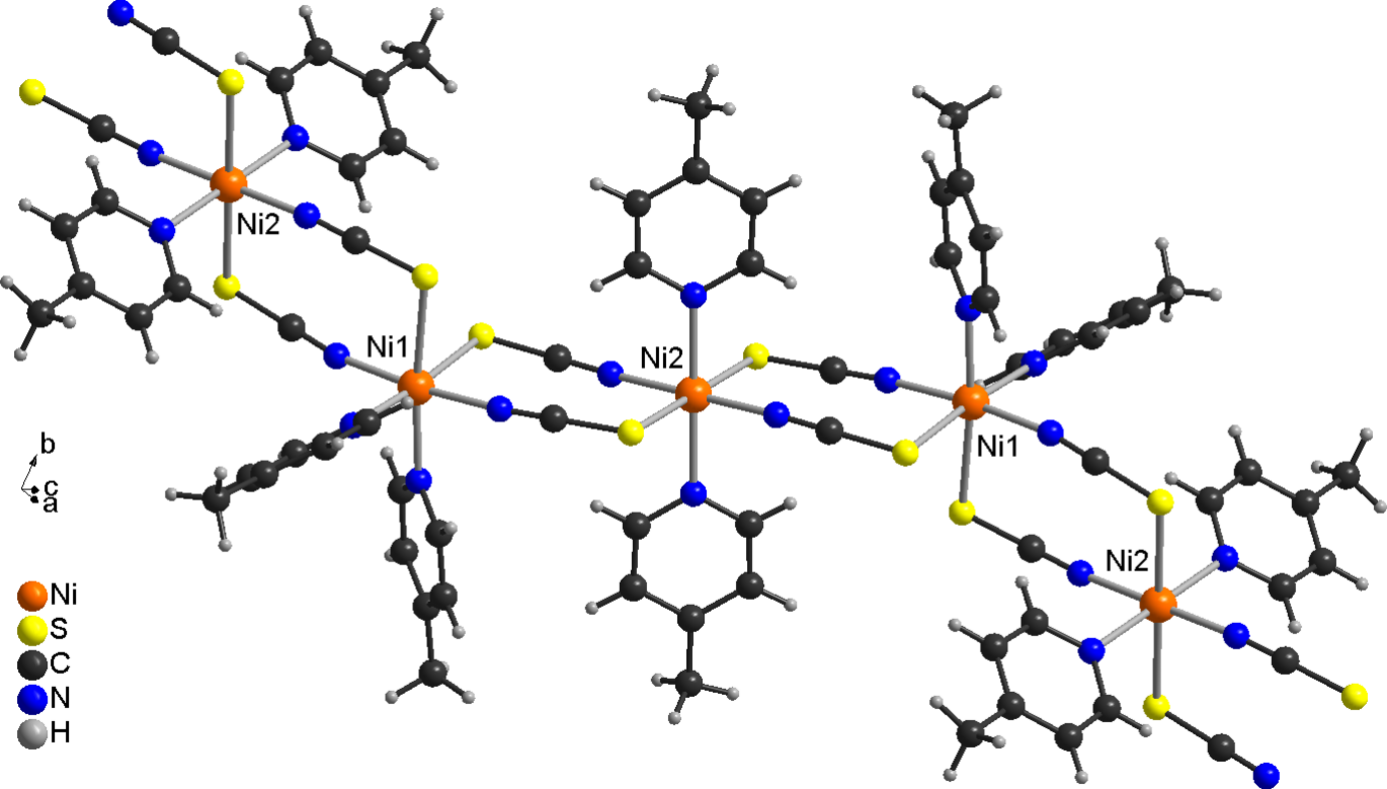
Crystal structure of the title compound with view of a chain and labeling of the Ni^II^ cations.

The asymmetric unit of the title compound, Ni(NCS)_2_(C_6_H_7_N)_2_, consists of two crystallographically independent Ni^II^ cations, two crystallographically independent thio­cyanate anions and two crystallographically independent 4-methyl­pyridine coligands (Fig. 2 ▸). Whereas the anionic and neutral ligands occupy general positions, one of the Ni^II^ cations (Ni1) is located on a crystallographic twofold rotation axis, and the second Ni^II^ cation (Ni2) occupies a center of inversion. Each of the Ni^II^ cations is sixfold coordinated by two N-bonding and two S-bonding μ-1,3-bridging thio­cyanate anions as well as two 4-methyl­pyridine coligands. Ni2 shows an all-*trans* configuration whereas Ni1 is in a *cis*–*cis–trans* arrangement with the S-bonding thio­cyanate anions and the 4-methyl­pyridine co­ligands in *cis* and the N-bonding thio­cyanate in *trans* positions (Fig. 3 ▸). For the Ni^II^ cation that shows an all-*trans* coordination, the Ni—S bond lengths are a bit shorter, whereas the Ni—N distances to the coligands are slightly longer compared to the cation in the *cis–cis–trans* configuration (Table 1 ▸). The metal cations are linked by pairs of μ-1,3-bridging anionic ligands into chains that, because of the alternating all-*trans* and *cis*–*cis*–*trans* configurations of the metal ions, are corrugated (Fig. 3 ▸).

This compound is isotypic to the thermodynamically stable isomer of Ni(NCS)_2_(4-chloro­pyridine)_2_ (UHUVIF01; Böhme *et al.*, 2020 ▸), which indicates that the title compound is thermodynamically stable. This is further supported by the fact that in all of our synthetic work we never found hints that a further isomer could be prepared.

## 3. Supra­molecular features

**Figure 4:**
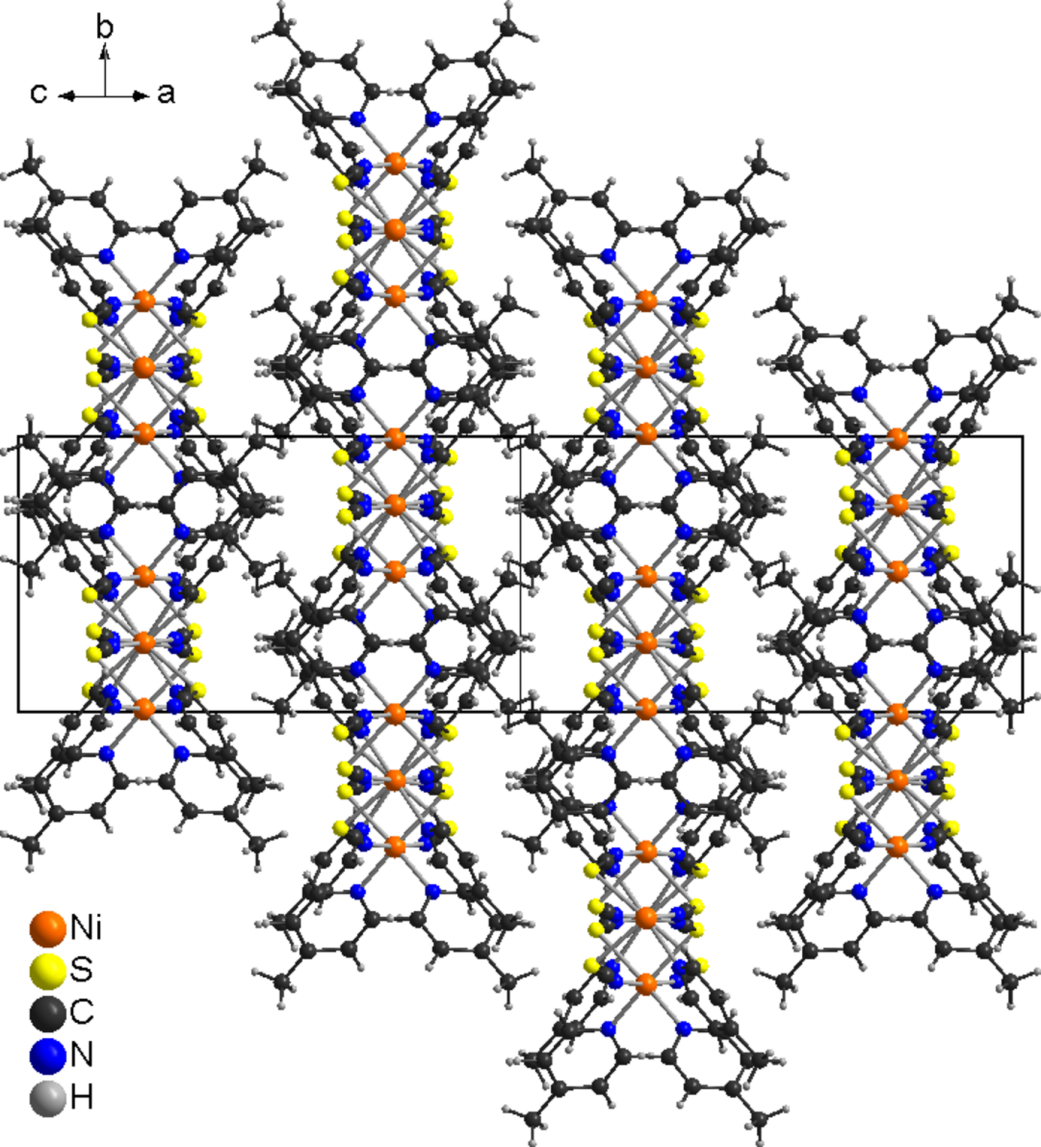
Crystal structure of the title compound with view along [101].

In the crystal structure of the title compound, the chains propagate along [101] with each chain surrounded by six neighboring chains (Fig. 4 ▸). There are no significant inter­molecular C—H⋯N or C—H⋯S contacts and there are also no hints of any π–π stacking inter­actions.

## 4. Database survey

A search in the CSD (version 5.43, last update December 2023; Groom *et al.*, 2016 ▸) using CONQUEST (Bruno *et al.*, 2002 ▸) for compounds based on Ni(NCS)_2_ and 4-methyl­pyridine revealed that several such compounds have already been reported. This include the two compounds Ni(NCS)_2_(C_6_H_7_N)_2_ [QQQGJV (Solaculu *et al.*, 1974 ▸) and ITMPNI (Lipowski & Andreetti, 1978 ▸)], already mentioned in the *Chemical context* section and one chain compound with the same composition that crystallizes as a *p*-toluidine solvate (CECDET; Micu-Semeniuc *et al.*, 1983 ▸). For the latter compound, no atomic coordinates are given. All remaining compounds consist of discrete complexes with an octa­hedral Ni coordination including Ni(NCS)_2_(C_6_H_7_N)_4_ [ICMPNI01 (Kerr & Williams, 1977 ▸); ICMPNI03 (Soldatov *et al.*, 2004 ▸); ICMPNI (Andreetti *et al.*, 1972 ▸); ICMPNI02 (Harris *et al.*, 2001 ▸); ICMPNI04 and ICMPNI05 (Soldatov *et al.*, 2004 ▸) and ICMPNI06 (Harris *et al.*, 2003 ▸)]. The majority of hits refer to clathrates of Ni(NCS)_2_(C_6_H_7_N)_4_, which are not listed in detail here.

At this point it is noted that this corresponds to a very rare coordination, because in most compounds with the general composition *M*(NCS)_2_*L*_2_ (*M* = metal cation, *L* = coligand) an all-*trans* coordination is found, which leads to the formation of linear chains (Rams *et al.*, 2017*a* ▸, 2020 ▸). Linear chains are also found for a *cis–cis–trans*-coordination, but only if the co­ligands are in the *trans*-position and the two N and two S-bonding thio­cyanate anions are in the *cis*-position. This is the case in the isotypic compounds *M*(NCS)_2_(4-benzoyl­pyridine)_2_ with *M* = Co, Ni [respectively, ODEYII (Rams *et al.*, 2017*b* ▸) and GIQQUV (Jochim *et al.*, 2018 ▸)] or Co(NCS)_2_(2,3-di­methyl­pyrazine-1,4-dioxide (refcode PEVZOG; Shi *et al.*, 2007 ▸). If the two bridging S-bonded thio­cyanate anions are in *trans*-positions as in, *e.g.* Ni(NCS)_2_(2,2′-bi­pyridine (GIQREG; Jochim *et al.*, 2018 ▸), or Mn(NCS)_2_(4-nitro­pyridine *N*-oxide (SINKUW; Shi *et al.*, 2006*a* ▸), the chains are corrugated. Corrugated chains are also observed if the two bridging N-bonded thio­cyanate anions are in *trans*-positions and this is the case *e.g.* in Ni(NCS)_2_[1-(2-amino­eth­yl)pyrrolidine-*N*,*N*′] (ABOBIC; Maji *et al.*, 2001 ▸). Finally, there are also examples for an all-*cis* configuration that also leads to the formation of corrugated chains and this includes *e.g.* Ni(NCS)_2_(4-methyl­pyridine *N*-oxide [PEDSUN (Shi *et al.*, 2006*b* ▸) and PEDSUN01 (Marsh, 2009 ▸)].

## 5. Additional investigations

**Figure 5:**
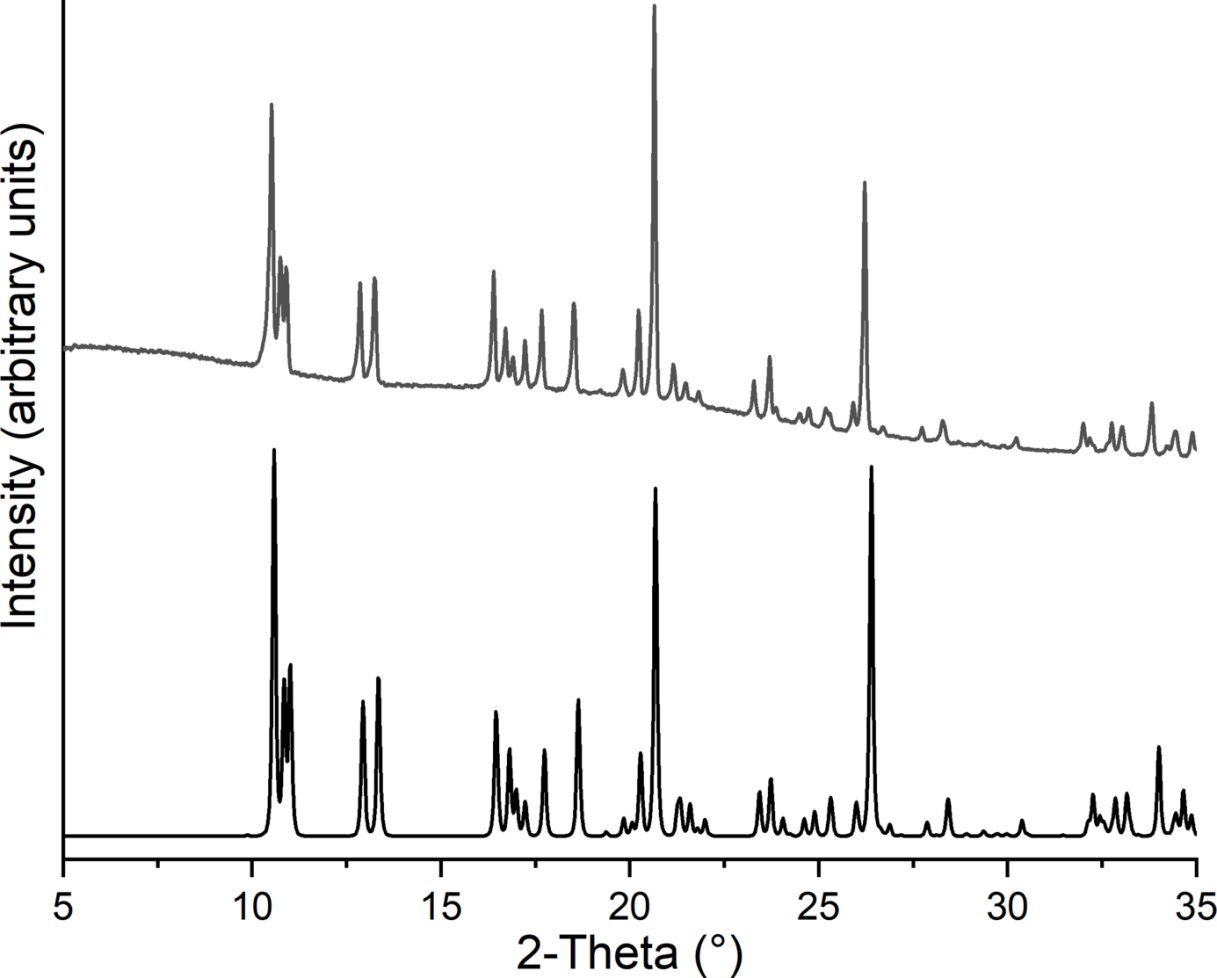
Experimental (top) and calculated (bottom) X-ray powder pattern of the title compound.

Powder X-ray diffraction measurements demonstrate that the title compound was obtained as a pure phase (Fig. 5 ▸).

The title compound was also investigated by thermogravimetry and differential thermoanalysis (TG-DTA) measurements. Upon heating, several mass losses are observed that are accompanied by endothermic events in the DTA curve (Fig. S1). From the DTG curve it is obvious that all mass losses are poorly resolved (Fig. S1). The experimental mass loss of the first and second steps is in rough agreement with that calculated for the removal of one 4-methyl­pyridine ligand in each step (Δ*m*_calc._ = 17.0%), indicating that a more 4-methyl­pyridine-deficient compound with the composition Ni(NCS)_2_(4-methyl­pyridine) has formed.

**Figure 6:**
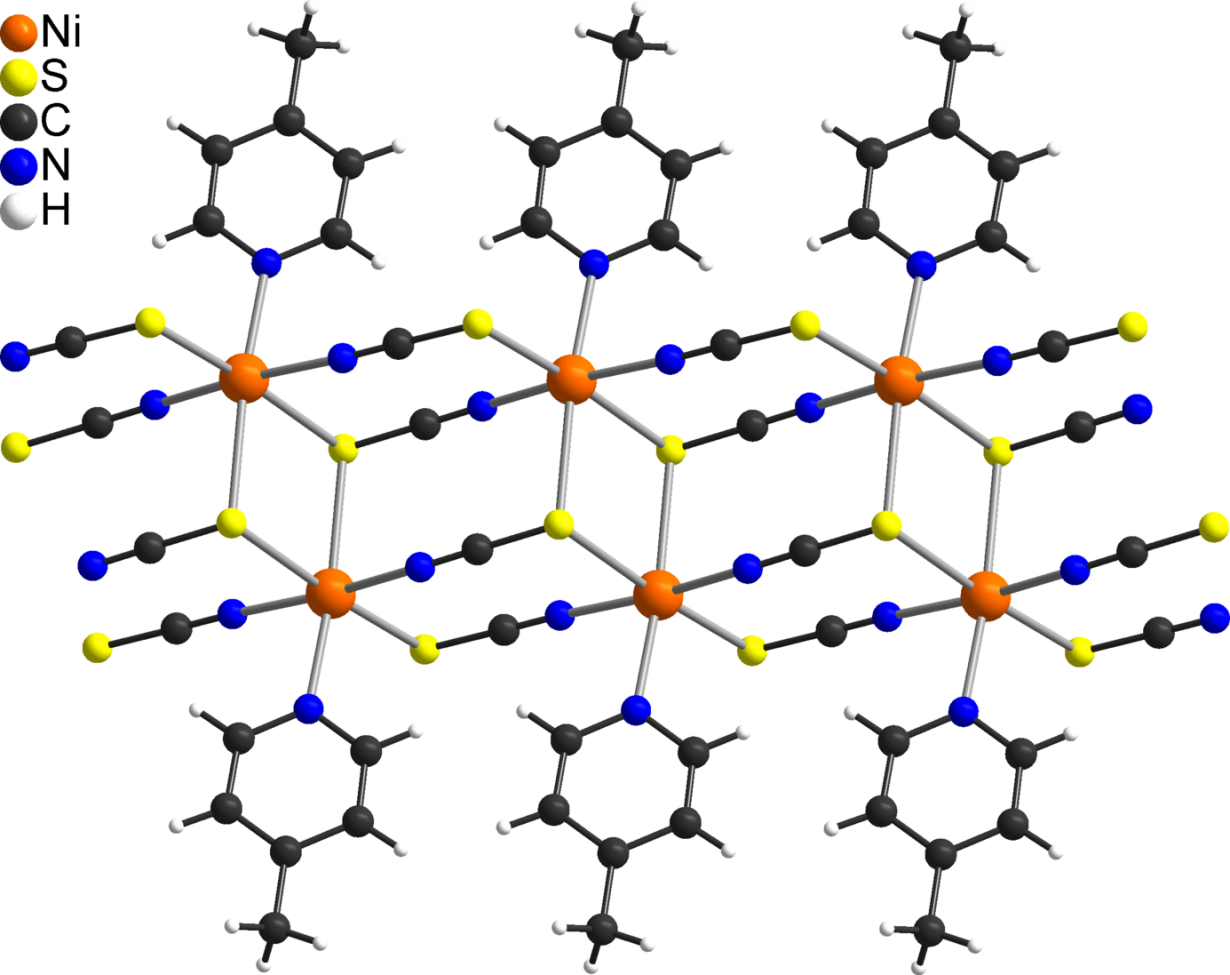
Crystal structure of Ni(NCS)_2_(C_6_H_7_N) obtained by thermal decomposition of the title compound.

Lowering the heating rate did not lead to better resolved curves and, therefore, isolation of this inter­mediate seems to be impossible. It was also not possible to prepare this phase from solution, even if an excess of Ni^II^ was used in the synthesis. Therefore, samples of the title compound were annealed for different times at different temperatures below the decomposition temperature observed in the TG-DTA measurements, until no reflections of the pristine compound **1** were present. In this case, a well-defined crystalline phase was obtained, for which the CN stretching vibrations of the anionic ligands are observed at 2118, 2141 and 2196 cm^−1^, indicating that a more complex thio­cyanate network has formed (Fig. S2). Unfortunately, indexing of this pattern did not lead to a reasonable unit cell but we reported the crystal structure of a compound with the composition Cd(NCS)_2_(C_6_H_7_N) a few years ago (Neumann *et al.*, 2020 ▸). Because Cd^II^ cations are much more chalcophilic than Ni^II^ cations, such compounds can easily be prepared and crystallized from solution. Based on the crystallographic data of Cd(NCS)_2_(C_6_H_7_N), a Rietveld refinement was performed for the residue obtained by thermal decomposition of the title compound, which proves that the Ni compound is isotypic and that this sample is contaminated with a small amount of Ni(NCS)_2_ [6.1 (3) wt.%], which might originate from a slightly too long tempering of the title compound (Fig. S3). In the crystal structure of Ni(NCS)_2_(C_6_H_7_N), the Ni^II^ cations are octa­hedrally coordinated by one 4-methyl­pyridine coligand, two N- and three S-bonding bridging thio­cyanate anions (Fig. 6 ▸). The metal cations are linked by one μ-1,3(*N*,*S*)- and one μ-1,3,3(*N*,*S*,*S*)-bridging thio­cyanate anion into single chains that condense *via* the μ-1,3,3(*N*,*S*,*S*)-bridging anionic ligands into double chains. The single chains are linked by each two S atoms sharing common edges, forming Ni_2_S_2_ rings.

Finally, it is noted that for the isotypic compounds with nickel and cobalt and 4-chloro­pyridine, no ligand-deficient compounds were detected and the reason for this difference in the thermal reactivity for isotypic compounds is not clear.

## 6. Synthesis and crystallization

**Synthesis**

4-Methyl­pyridine (purity 98%) and KNCS were obtained from Sigma-Aldrich and NiCl_2_·6H_2_O was purchased from Carl Roth.

6.00 mmol (1471 mg) of NiCl_2_·6H_2_O, 12.00 mmol (1160 mg) of KNCS and 12.00 mmol (1160 µl) of 4-methyl­pyridine were stirred in 5 ml of demineralized water at room temperature for 1 day. The precipitate was filtered off, washed with demineralized water and dried in air. Single crystals of the title compound were obtained using the same molar ratio of the reactants without stirring. Elemental analysis for C_14_H_14_N_4_NiS_2_: calculated C 46.57, H 3.91, N 15.51; S 17.76, found C 46.78, H 4.08, N 15.72 S 17.94. The purity was also shown by powder X-ray diffraction (see Fig. S4). An IR spectrum of the title compound can be found in Fig. S4.

**Experimental details**

Elemental analysis was performed with a vario MICRO cube from Elementar Analysensysteme GmbH. IR spectra were recorded at room temperature on a Bruker Vertex70 FT-IR spectrometer using a broadband spectral range extension VERTEX FM for full mid and far IR. Thermogravimetry and differential thermoanalysis (TG–DTA) measurements were performed in a dynamic nitro­gen atmosphere in Al_2_O_3_ crucibles with an 8°C min^−1^ heating rate using a STA-PT 1000 thermobalance from Linseis. The TG–DTA instrument was calibrated using standard reference materials. X-ray powder diffraction experiments were performed using a Stoe STADI P transmission powder diffractometer with Cu *K*α1 radiation (λ = 1.540598 Å), a Johann-type Ge(111) monochromator and a MYTHEN 1K detector from Dectris. The structure refinement was carried out using *TOPAS* Academic version 6.0 (Coelho, 2018 ▸). For the Rietveld refinement, the structure of Cd(NCS)_2_(C_6_H_7_N) was used as starting model with Cd replaced by Ni. All ligands were set up as rigid bodies with bond lengths taken from the literature, including split positions for four carbon atoms to model rotational disorder of the ligand. The positions of the cation and all ligands were subject to unconstrained refinement, which quickly converged to a convincing *R*_Bragg_ of 1.2%.

## 7. Refinement

Crystal data, data collection and structure refinement details are summarized in Table 2 ▸. The hydrogen atoms were positioned with idealized geometry (methyl H atoms allowed to rotate but not to tip) and were refined with *U*_iso_(H) = 1.2*U*_eq_(C) (1.5 for methyl H atoms) using a riding model.

## Supplementary Material

Crystal structure: contains datablock(s) I. DOI: 10.1107/S2056989024005887/hb8101sup1.cif

Structure factors: contains datablock(s) I. DOI: 10.1107/S2056989024005887/hb8101Isup2.hkl

Figure S1. DTG, TG and DTA curve for the title compound, measured with 4C/min. Given is the mass loss in % and the peak temperature in C. DOI: 10.1107/S2056989024005887/hb8101sup3.png

Figure S2. IR spectrum of the residue obtained by thermal annealing of the title compound. Given are the values for the CN stretching vibrations. DOI: 10.1107/S2056989024005887/hb8101sup4.png

Figure S3. Difference plot for the final Rietveld refinement of Ni(NCS)2(4-methylpyridine), rwp = 3.8 %, rBragg = 1.2 %. DOI: 10.1107/S2056989024005887/hb8101sup5.png

Figure S4. IR spectrum of the title compound. Given is the value for the CN stretching vibration. DOI: 10.1107/S2056989024005887/hb8101sup6.png

CCDC reference: 2363246

Additional supporting information:  crystallographic information; 3D view; checkCIF report

## Figures and Tables

**Table 1:** Selected geometric parameters (Å, °)

Ni1—N1	2.0358 (17)	Ni2—S1	2.5208 (5)
Ni1—S2	2.5653 (6)	Ni2—N2	2.0261 (17)
Ni1—N11	2.0956 (18)	Ni2—N21	2.1345 (18)
			
N1—Ni1—N1^i^	174.18 (10)	S1—Ni2—S1^ii^	180.0
S2—Ni1—S2^i^	89.50 (3)	N2—Ni2—N2^ii^	180.0
N11—Ni1—N11^i^	89.52 (9)	N21—Ni2—N21^ii^	180.0

Symmetry codes: (i) 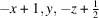; (ii) 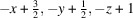.

**Table 2:** Experimental details

Crystal data	
Chemical formula	[Ni(NCS)_2_(C_6_H_7_N)_2_]
*M*_r_	361.12
Crystal system, space group	Monoclinic, *C*2/*c*
Temperature (K)	220
*a*, *b*, *c* (Å)	20.0352 (12), 9.1536 (5), 19.2268 (12)
β (°)	116.783 (6)
*V* (Å^3^)	3147.8 (4)
*Z*	8
Radiation type	Mo *K*α
μ (mm^−1^)	1.50
Crystal size (mm)	0.16 × 0.12 × 0.08

Data collection	
Diffractometer	Stoe IPDS2
Absorption correction	Numerical (*X-RED* and *X-SHAPE*; Stoe, 2008 ▸)
*T*_min_, *T*_max_	0.691, 0.864
No. of measured, independent and observed [*I* > 2σ(*I*)] reflections	15838, 3805, 3113
*R*_int_	0.035
(sin θ/λ)_max_ (Å^−1^)	0.660

Refinement	
*R*[*F*^2^ > 2σ(*F*^2^)], *wR*(*F*^2^), *S*	0.034, 0.091, 1.03
No. of reflections	3805
No. of parameters	195
H-atom treatment	H-atom parameters constrained
Δρ_max_, Δρ_min_ (e Å^−3^)	0.48, −0.47

Computer programs: *X-AREA* (Stoe, 2008 ▸), *SHELXT2014/4* (Sheldrick, 2015*a* ▸), *SHELXL2016/6* (Sheldrick, 2015*b* ▸), *DIAMOND* (Brandenburg & Putz, 1999 ▸) and *XP* in *SHELXTL*-PC (Sheldrick, 2008 ▸) and *publCIF* (Westrip, 2010 ▸).

## supplementary crystallographic information

### catena-Poly[[bis(4-methylpyridine-κN)nickel(II)]-di-µ-thiocyanato-κ2N:S;κ2S:N] Crystal data

**Table:**

[Ni(NCS)_2_(C_6_H_7_N)_2_]	*F*(000) = 1488
*M**_r_* = 361.12	*D*_x_ = 1.524 Mg m^−^^3^
Monoclinic, *C*2/*c*	Mo *K*α radiation, λ = 0.71073 Å
*a* = 20.0352 (12) Å	Cell parameters from 7998 reflections
*b* = 9.1536 (5) Å	θ = 2.4–28.0°
*c* = 19.2268 (12) Å	µ = 1.50 mm^−^^1^
β = 116.783 (6)°	*T* = 220 K
*V* = 3147.8 (4) Å^3^	Block, green
*Z* = 8	0.16 × 0.12 × 0.08 mm

### catena-Poly[[bis(4-methylpyridine-κN)nickel(II)]-di-µ-thiocyanato-κ2N:S;κ2S:N] Data collection

**Table:**

Stoe IPDS-2 diffractometer	3113 reflections with *I* > 2σ(*I*)
ω scans	*R*_int_ = 0.035
Absorption correction: numerical (X-Red and X-Shape; Stoe, 2008)	θ_max_ = 28.0°, θ_min_ = 2.4°
*T*_min_ = 0.691, *T*_max_ = 0.864	*h* = −26→26
15838 measured reflections	*k* = −12→12
3805 independent reflections	*l* = −25→25

### catena-Poly[[bis(4-methylpyridine-κN)nickel(II)]-di-µ-thiocyanato-κ2N:S;κ2S:N] Refinement

**Table:**

Refinement on *F*^2^	Hydrogen site location: inferred from neighbouring sites
Least-squares matrix: full	H-atom parameters constrained
*R*[*F*^2^ > 2σ(*F*^2^)] = 0.034	*w* = 1/[σ^2^(*F*_o_^2^) + (0.0616*P*)^2^ + 0.1916*P*] where *P* = (*F*_o_^2^ + 2*F*_c_^2^)/3
*wR*(*F*^2^) = 0.091	(Δ/σ)_max_ = 0.001
*S* = 1.03	Δρ_max_ = 0.48 e Å^−^^3^
3805 reflections	Δρ_min_ = −0.47 e Å^−^^3^
195 parameters	Extinction correction: *SHELXL2016/6* (Sheldrick, 2015b), Fc^*^=kFc[1+0.001xFc^2^λ^3^/sin(2θ)]^-1/4^
0 restraints	Extinction coefficient: 0.0034 (3)

### catena-Poly[[bis(4-methylpyridine-κN)nickel(II)]-di-µ-thiocyanato-κ2N:S;κ2S:N] Special details

**Table:**

Geometry. All esds (except the esd in the dihedral angle between two l.s. planes) are estimated using the full covariance matrix. The cell esds are taken into account individually in the estimation of esds in distances, angles and torsion angles; correlations between esds in cell parameters are only used when they are defined by crystal symmetry. An approximate (isotropic) treatment of cell esds is used for estimating esds involving l.s. planes.

### catena-Poly[[bis(4-methylpyridine-κN)nickel(II)]-di-µ-thiocyanato-κ2N:S;κ2S:N] Fractional atomic coordinates and isotropic or equivalent isotropic displacement parameters (Å^2^)

**Table:**

	*x*	*y*	*z*	*U*_iso_*/*U*_eq_	
Ni1	0.500000	0.49095 (4)	0.250000	0.02030 (11)	
Ni2	0.750000	0.250000	0.500000	0.02012 (11)	
S1	0.66327 (3)	0.42321 (6)	0.52157 (3)	0.02554 (14)	
C1	0.60061 (10)	0.4565 (2)	0.43118 (11)	0.0199 (4)	
N1	0.55720 (9)	0.4797 (2)	0.36824 (10)	0.0240 (4)	
S2	0.58017 (3)	0.29192 (7)	0.23054 (3)	0.03120 (15)	
C2	0.64768 (11)	0.2743 (2)	0.31946 (11)	0.0213 (4)	
N2	0.69454 (10)	0.2631 (2)	0.38228 (10)	0.0256 (4)	
N11	0.57268 (9)	0.6535 (2)	0.24781 (9)	0.0230 (3)	
C11	0.59509 (11)	0.6616 (2)	0.19194 (11)	0.0260 (4)	
H11	0.576619	0.592325	0.151505	0.031*	
C12	0.64407 (12)	0.7668 (2)	0.19100 (12)	0.0271 (4)	
H12	0.657378	0.769083	0.149963	0.033*	
C13	0.67375 (11)	0.8695 (2)	0.25052 (12)	0.0264 (4)	
C14	0.64970 (13)	0.8611 (3)	0.30801 (13)	0.0332 (5)	
H14	0.667379	0.928794	0.349211	0.040*	
C15	0.60000 (13)	0.7538 (3)	0.30461 (13)	0.0308 (5)	
H15	0.584526	0.750894	0.344048	0.037*	
C16	0.72901 (14)	0.9821 (3)	0.25378 (16)	0.0391 (6)	
H16A	0.758663	0.943774	0.229594	0.059*	
H16B	0.761580	1.006536	0.307691	0.059*	
H16C	0.702696	1.069001	0.226168	0.059*	
N21	0.68081 (9)	0.0707 (2)	0.49738 (10)	0.0246 (4)	
C21	0.70854 (12)	−0.0404 (3)	0.54745 (13)	0.0313 (5)	
H21	0.760343	−0.043195	0.579938	0.038*	
C22	0.66472 (13)	−0.1516 (3)	0.55383 (13)	0.0343 (5)	
H22	0.686953	−0.227367	0.590015	0.041*	
C23	0.58782 (12)	−0.1514 (3)	0.50670 (13)	0.0286 (4)	
C24	0.55988 (12)	−0.0385 (3)	0.45291 (13)	0.0298 (5)	
H24	0.508574	−0.034966	0.418356	0.036*	
C25	0.60707 (11)	0.0684 (3)	0.44994 (12)	0.0277 (4)	
H25	0.586557	0.143308	0.412862	0.033*	
C26	0.53820 (14)	−0.2658 (3)	0.51501 (16)	0.0367 (5)	
H26A	0.548385	−0.359533	0.498245	0.055*	
H26B	0.486273	−0.239712	0.483032	0.055*	
H26C	0.547883	−0.272090	0.569101	0.055*	

### catena-Poly[[bis(4-methylpyridine-κN)nickel(II)]-di-µ-thiocyanato-κ2N:S;κ2S:N] Atomic displacement parameters (Å^2^)

**Table:**

	*U*^11^	*U*^22^	*U*^33^	*U*^12^	*U*^13^	*U*^23^
Ni1	0.01658 (17)	0.0244 (2)	0.01412 (17)	0.000	0.00174 (13)	0.000
Ni2	0.01671 (17)	0.0250 (2)	0.01461 (17)	0.00380 (13)	0.00348 (13)	0.00186 (12)
S1	0.0231 (2)	0.0337 (3)	0.0144 (2)	0.00884 (19)	0.00373 (17)	0.00084 (18)
C1	0.0179 (8)	0.0198 (9)	0.0215 (9)	0.0000 (7)	0.0084 (7)	−0.0027 (7)
N1	0.0205 (7)	0.0295 (10)	0.0168 (8)	0.0044 (6)	0.0039 (6)	0.0002 (6)
S2	0.0295 (3)	0.0368 (3)	0.0162 (2)	0.0087 (2)	0.00049 (19)	−0.00435 (19)
C2	0.0220 (8)	0.0213 (10)	0.0218 (9)	0.0017 (7)	0.0109 (7)	−0.0009 (7)
N2	0.0235 (8)	0.0316 (10)	0.0179 (8)	0.0068 (7)	0.0060 (6)	0.0016 (6)
N11	0.0201 (7)	0.0262 (9)	0.0215 (7)	−0.0024 (6)	0.0084 (6)	−0.0045 (6)
C11	0.0241 (9)	0.0308 (11)	0.0211 (9)	−0.0018 (8)	0.0085 (7)	−0.0070 (8)
C12	0.0280 (9)	0.0313 (11)	0.0254 (10)	−0.0007 (8)	0.0149 (8)	−0.0028 (8)
C13	0.0229 (9)	0.0270 (11)	0.0296 (10)	−0.0005 (8)	0.0121 (8)	−0.0025 (8)
C14	0.0373 (11)	0.0352 (13)	0.0303 (10)	−0.0109 (10)	0.0182 (9)	−0.0137 (9)
C15	0.0354 (11)	0.0333 (12)	0.0284 (10)	−0.0082 (9)	0.0187 (9)	−0.0121 (8)
C16	0.0417 (13)	0.0357 (14)	0.0477 (15)	−0.0113 (10)	0.0272 (12)	−0.0077 (10)
N21	0.0210 (7)	0.0274 (10)	0.0236 (8)	0.0025 (6)	0.0084 (6)	0.0006 (6)
C21	0.0234 (9)	0.0334 (12)	0.0304 (11)	0.0012 (9)	0.0062 (8)	0.0051 (9)
C22	0.0353 (11)	0.0305 (12)	0.0308 (11)	0.0021 (9)	0.0091 (9)	0.0054 (9)
C23	0.0318 (10)	0.0273 (11)	0.0308 (10)	−0.0014 (8)	0.0177 (8)	−0.0071 (8)
C24	0.0224 (9)	0.0336 (12)	0.0316 (11)	0.0018 (8)	0.0105 (8)	−0.0036 (9)
C25	0.0232 (9)	0.0317 (12)	0.0261 (10)	0.0037 (8)	0.0094 (8)	0.0006 (8)
C26	0.0397 (12)	0.0333 (13)	0.0450 (13)	−0.0061 (10)	0.0260 (11)	−0.0074 (10)

### catena-Poly[[bis(4-methylpyridine-κN)nickel(II)]-di-µ-thiocyanato-κ2N:S;κ2S:N] Geometric parameters (Å, º)

**Table:**

Ni1—N1	2.0358 (17)	C13—C14	1.393 (3)
Ni1—N1^i^	2.0358 (17)	C13—C16	1.493 (3)
Ni1—S2^i^	2.5653 (6)	C14—H14	0.9400
Ni1—S2	2.5653 (6)	C14—C15	1.379 (3)
Ni1—N11^i^	2.0955 (17)	C15—H15	0.9400
Ni1—N11	2.0956 (18)	C16—H16A	0.9700
Ni2—S1^ii^	2.5208 (5)	C16—H16B	0.9700
Ni2—S1	2.5208 (5)	C16—H16C	0.9700
Ni2—N2	2.0261 (17)	N21—C21	1.337 (3)
Ni2—N2^ii^	2.0262 (17)	N21—C25	1.342 (3)
Ni2—N21	2.1345 (18)	C21—H21	0.9400
Ni2—N21^ii^	2.1345 (18)	C21—C22	1.385 (3)
S1—C1	1.6501 (19)	C22—H22	0.9400
C1—N1	1.148 (3)	C22—C23	1.392 (3)
S2—C2	1.6414 (19)	C23—C24	1.389 (3)
C2—N2	1.153 (3)	C23—C26	1.501 (3)
N11—C11	1.340 (3)	C24—H24	0.9400
N11—C15	1.340 (3)	C24—C25	1.380 (3)
C11—H11	0.9400	C25—H25	0.9400
C11—C12	1.381 (3)	C26—H26A	0.9700
C12—H12	0.9400	C26—H26B	0.9700
C12—C13	1.390 (3)	C26—H26C	0.9700
			
N1—Ni1—N1^i^	174.18 (10)	C11—C12—H12	119.9
N1^i^—Ni1—S2	82.84 (5)	C11—C12—C13	120.2 (2)
N1—Ni1—S2^i^	82.84 (5)	C13—C12—H12	119.9
N1^i^—Ni1—S2^i^	93.01 (5)	C12—C13—C14	116.3 (2)
N1—Ni1—S2	93.01 (5)	C12—C13—C16	122.4 (2)
N1—Ni1—N11^i^	93.45 (7)	C14—C13—C16	121.3 (2)
N1^i^—Ni1—N11^i^	90.68 (7)	C13—C14—H14	119.9
N1—Ni1—N11	90.68 (7)	C15—C14—C13	120.1 (2)
N1^i^—Ni1—N11	93.45 (7)	C15—C14—H14	119.9
S2—Ni1—S2^i^	89.50 (3)	N11—C15—C14	123.2 (2)
N11^i^—Ni1—S2^i^	90.86 (5)	N11—C15—H15	118.4
N11—Ni1—S2	90.85 (5)	C14—C15—H15	118.4
N11^i^—Ni1—S2	173.52 (5)	C13—C16—H16A	109.5
N11—Ni1—S2^i^	173.52 (5)	C13—C16—H16B	109.5
N11—Ni1—N11^i^	89.52 (9)	C13—C16—H16C	109.5
S1—Ni2—S1^ii^	180.0	H16A—C16—H16B	109.5
N2—Ni2—S1^ii^	85.71 (5)	H16A—C16—H16C	109.5
N2^ii^—Ni2—S1	85.71 (5)	H16B—C16—H16C	109.5
N2—Ni2—S1	94.29 (5)	C21—N21—Ni2	120.87 (14)
N2^ii^—Ni2—S1^ii^	94.29 (5)	C21—N21—C25	116.78 (19)
N2—Ni2—N2^ii^	180.0	C25—N21—Ni2	122.18 (15)
N2^ii^—Ni2—N21	90.24 (7)	N21—C21—H21	118.4
N2—Ni2—N21^ii^	90.23 (7)	N21—C21—C22	123.25 (19)
N2—Ni2—N21	89.76 (7)	C22—C21—H21	118.4
N2^ii^—Ni2—N21^ii^	89.77 (7)	C21—C22—H22	120.0
N21^ii^—Ni2—S1^ii^	89.95 (5)	C21—C22—C23	120.1 (2)
N21^ii^—Ni2—S1	90.05 (5)	C23—C22—H22	120.0
N21—Ni2—S1^ii^	90.05 (5)	C22—C23—C26	121.4 (2)
N21—Ni2—S1	89.95 (5)	C24—C23—C22	116.3 (2)
N21—Ni2—N21^ii^	180.0	C24—C23—C26	122.3 (2)
C1—S1—Ni2	101.33 (7)	C23—C24—H24	119.8
N1—C1—S1	179.8 (2)	C25—C24—C23	120.31 (19)
C1—N1—Ni1	164.70 (17)	C25—C24—H24	119.8
C2—S2—Ni1	101.18 (7)	N21—C25—C24	123.2 (2)
N2—C2—S2	179.1 (2)	N21—C25—H25	118.4
C2—N2—Ni2	162.45 (17)	C24—C25—H25	118.4
C11—N11—Ni1	122.82 (14)	C23—C26—H26A	109.5
C11—N11—C15	117.01 (18)	C23—C26—H26B	109.5
C15—N11—Ni1	120.16 (14)	C23—C26—H26C	109.5
N11—C11—H11	118.5	H26A—C26—H26B	109.5
N11—C11—C12	123.06 (19)	H26A—C26—H26C	109.5
C12—C11—H11	118.5	H26B—C26—H26C	109.5
			
Ni1—N11—C11—C12	−178.77 (16)	C15—N11—C11—C12	0.0 (3)
Ni1—N11—C15—C14	178.07 (19)	C16—C13—C14—C15	−178.4 (2)
Ni2—N21—C21—C22	173.01 (19)	N21—C21—C22—C23	0.0 (4)
Ni2—N21—C25—C24	−172.91 (17)	C21—N21—C25—C24	2.4 (3)
N11—C11—C12—C13	1.2 (3)	C21—C22—C23—C24	2.3 (3)
C11—N11—C15—C14	−0.8 (3)	C21—C22—C23—C26	−176.7 (2)
C11—C12—C13—C14	−1.7 (3)	C22—C23—C24—C25	−2.2 (3)
C11—C12—C13—C16	177.7 (2)	C23—C24—C25—N21	−0.1 (4)
C12—C13—C14—C15	1.0 (3)	C25—N21—C21—C22	−2.4 (3)
C13—C14—C15—N11	0.2 (4)	C26—C23—C24—C25	176.8 (2)

Symmetry codes: (i) −*x*+1, *y*, −*z*+1/2; (ii) −*x*+3/2, −*y*+1/2, −*z*+1.
