# Supplementary figures and images for: Synthesis, crystal structure and thermal properties of catena-poly[[bis­(4-methyl­pyridine)­nickel(II)]-di-μ-thio­cyanato], which shows an alternating all-trans and cis–cis–trans-coordination of the NiS2Np2Nt2 octa­hedra (p = 4-methyl­pyridine, t = thio­cyanate)
Source: Acta Crystallogr E Crystallogr Commun. 2024 Jun 21;80(Pt 7):771–6. doi: 10.1107/S2056989024005887 (PMC11223700; doi:10.1107/S2056989024005887)

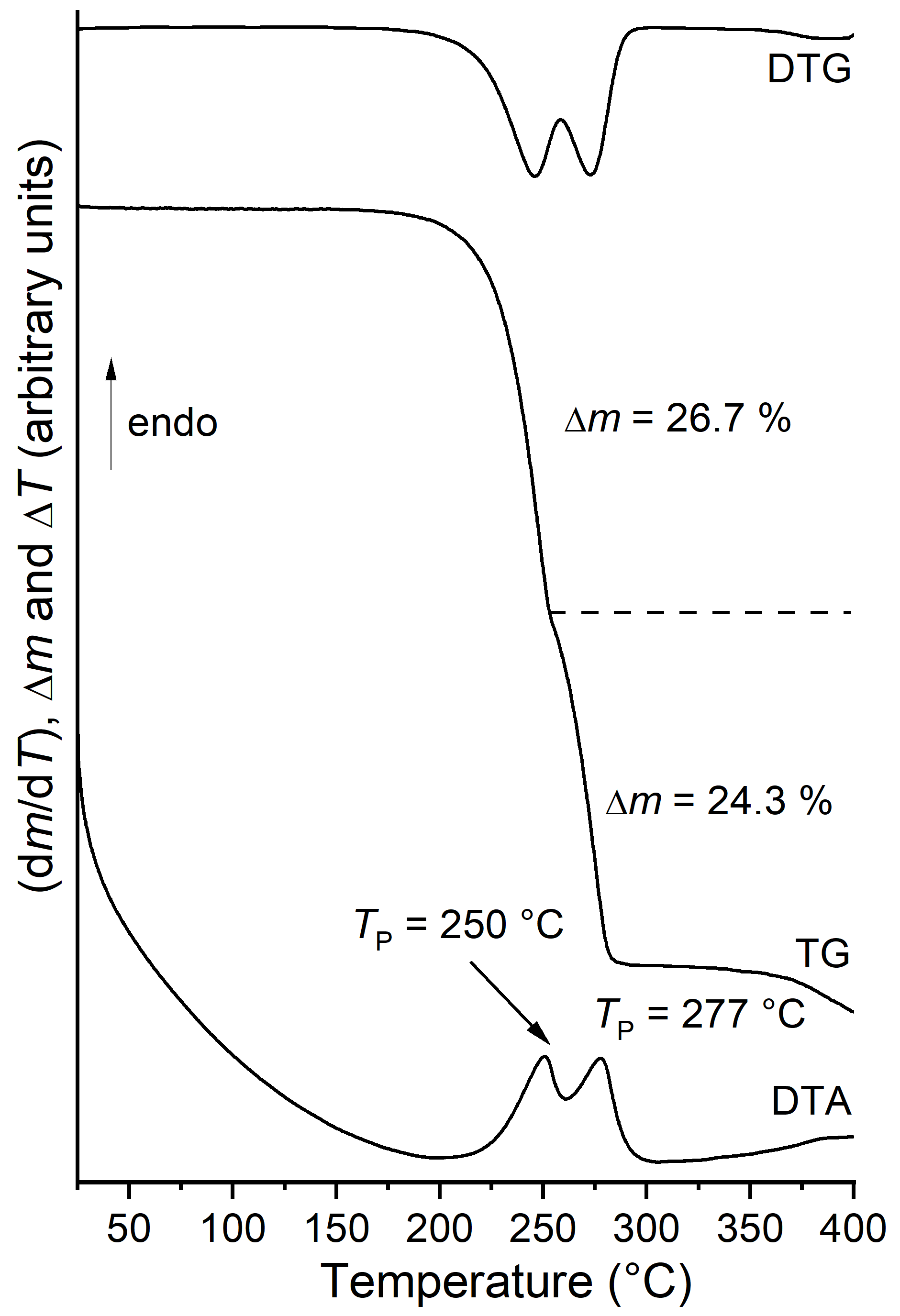

Supplement: Supplementary file 3 [file e-80-00771-sup3.png]

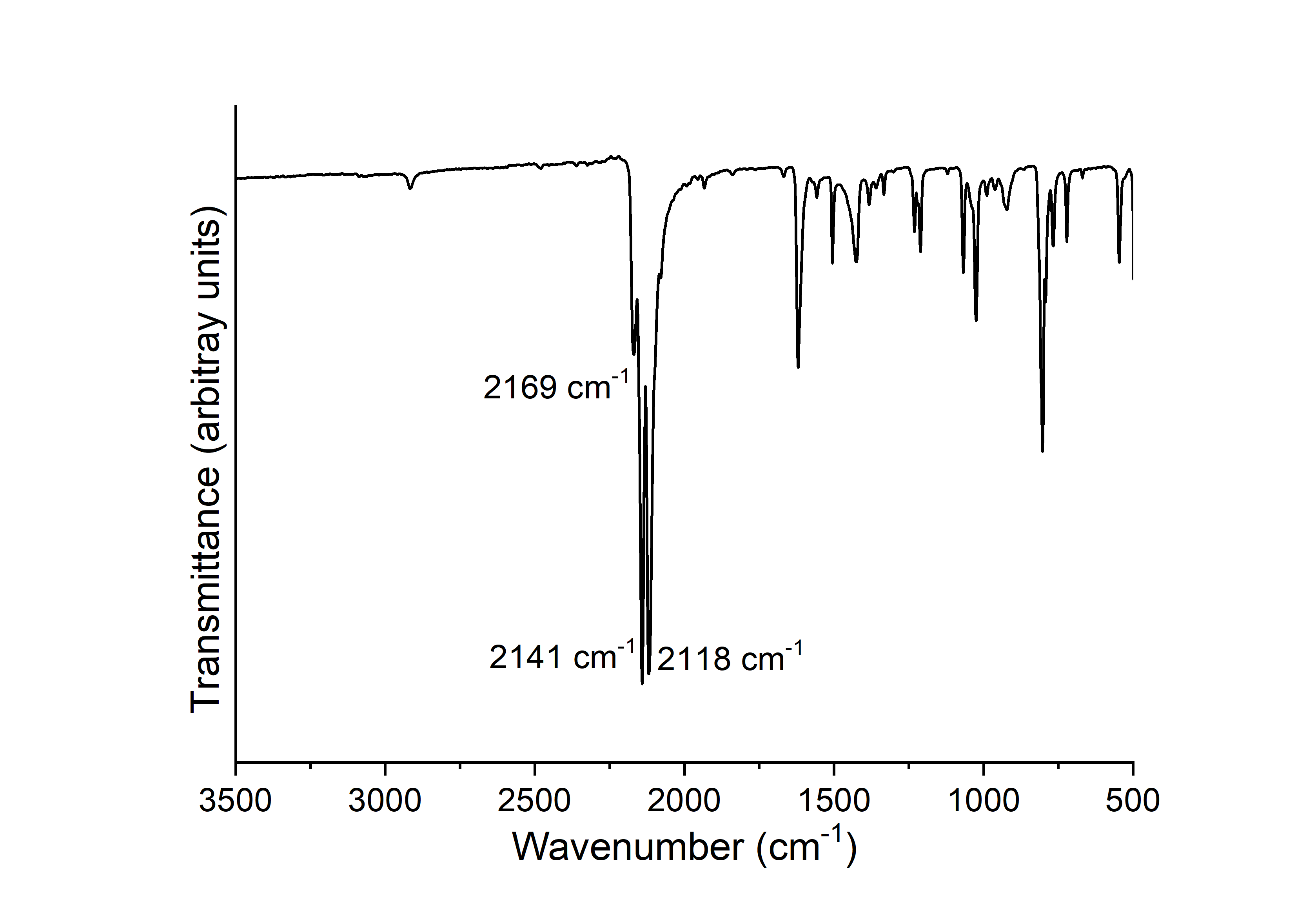

Supplement: Supplementary file 4 [file e-80-00771-sup4.png]

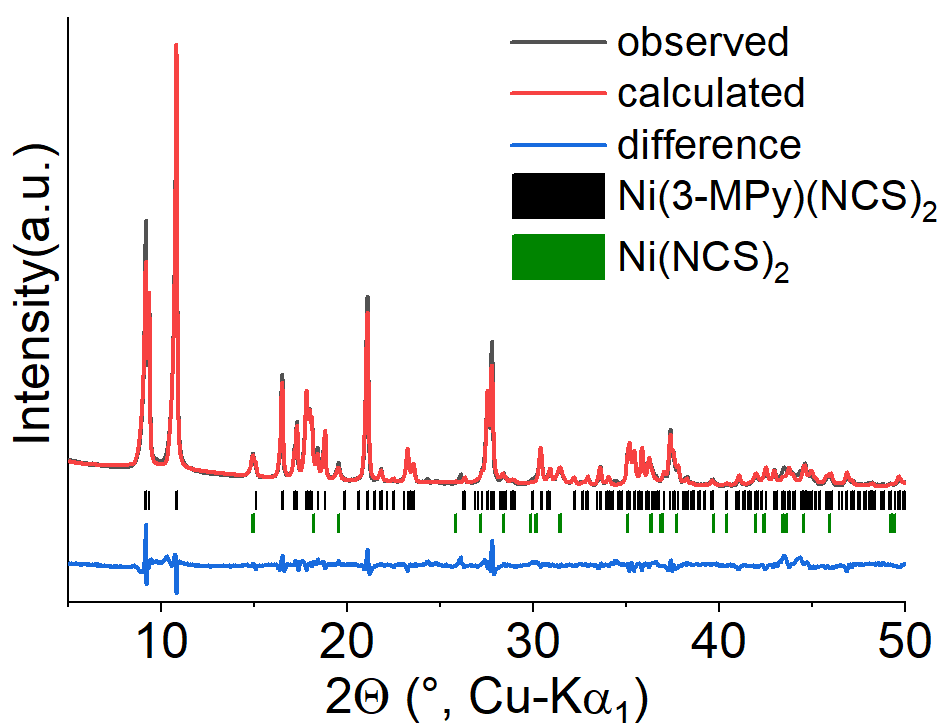

Supplement: Supplementary file 5 [file e-80-00771-sup5.png]

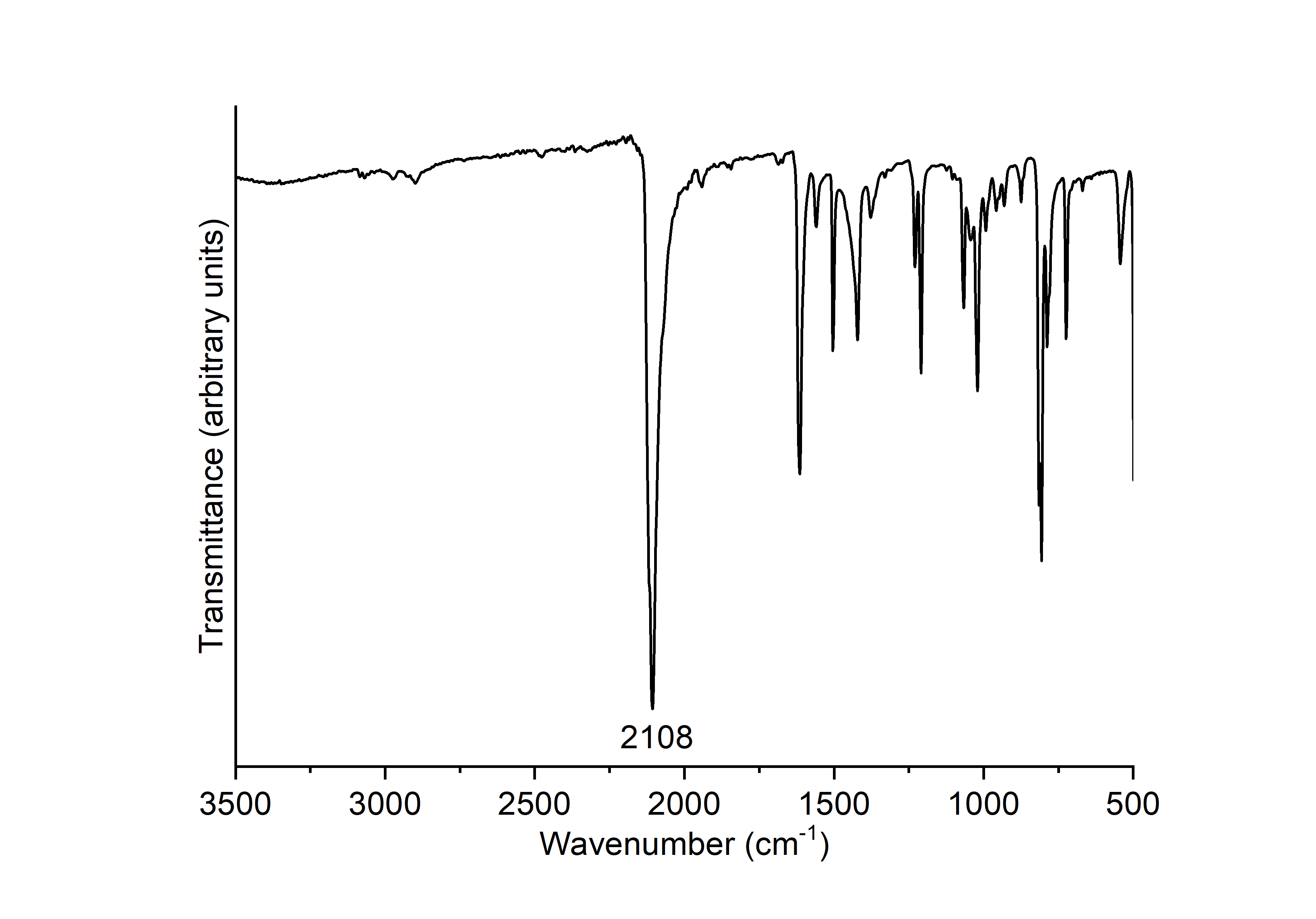

Supplement: Supplementary file 6 [file e-80-00771-sup6.png]
